# Guanylate-Binding Protein-Dependent Noncanonical Inflammasome Activation Prevents Burkholderia thailandensis-Induced Multinucleated Giant Cell Formation

**DOI:** 10.1128/mBio.02054-21

**Published:** 2021-08-17

**Authors:** Marisa Dilucca, Saray Ramos, Kateryna Shkarina, José Carlos Santos, Petr Broz

**Affiliations:** a Department of Biochemistry, University of Lausanne, Epalinges, Switzerland; University of Geneva

**Keywords:** antimicrobial mechanisms, guanylate-binding proteins, host-pathogen interactions, inflammasomes, innate immunity

## Abstract

Inflammasomes are cytosolic multiprotein signaling complexes that are activated upon pattern recognition receptor-mediated recognition of pathogen-derived ligands or endogenous danger signals. Their assembly activates the downstream inflammatory caspase-1 and caspase-4/5 (human) or caspase-11 (mouse), which induces cytokine release and pyroptotic cell death through the cleavage of the pore-forming effector gasdermin D. Pathogen detection by host cells also results in the production and release of interferons (IFNs), which fine-tune inflammasome-mediated responses. IFN-induced guanylate-binding proteins (GBPs) have been shown to control the activation of the noncanonical inflammasome by recruiting caspase-4 on the surface of cytosolic Gram-negative bacteria and promoting its interaction with lipopolysaccharide (LPS). The Gram-negative opportunistic bacterial pathogen Burkholderia thailandensis infects epithelial cells and macrophages and hijacks the host actin polymerization machinery to spread into neighboring cells. This process causes host cell fusion and the formation of so-called multinucleated giant cells (MNGCs). Caspase-1- and IFN-regulated caspase-11-mediated inflammasome pathways play an important protective role against B. thailandensis in mice, but little is known about the role of IFNs and inflammasomes during B. thailandensis infection of human cells, particularly epithelial cells. Here, we report that IFN-γ priming of human epithelial cells restricts B. thailandensis-induced MNGC formation in a GBP1-dependent manner. Mechanistically, GBP1 does not promote bacteriolysis or impair actin-based bacterial motility but acts by inducing caspase-4-dependent pyroptosis of the infected cell. In addition, we show that IFN-γ priming of human primary macrophages confers a more efficient antimicrobial effect through inflammasome activation, further confirming the important role that interferon signaling plays in restricting *Burkholderia* replication and spread.

## INTRODUCTION

*Burkholderia* is a genus of Gram-negative bacteria and includes species that are pathogenic for humans, such as Burkholderia mallei and the soil-dwelling species B. pseudomallei. The former is the causative agent of glanders, a contagious zoonotic infectious disease that primarily affects horses, whereas the latter is the etiological agent of human melioidosis (1–4). Melioidosis is an infectious disease present mostly in Asia, Africa, and South America and endemic in Thailand and northern Australia. The disease is thought to develop upon bacterial infection through inhalation, ingestion of contaminated food or water, or direct contact with the soil through skin abrasions. Depending on the infection route, patients display a wide range of clinical signs and symptoms (e.g., sepsis, pneumonia, and encephalitis, etc.) that can lead to a fatal outcome, mostly if left untreated (1–4). B. pseudomallei is naturally resistant to several antibiotics, and its wide environmental dissemination and ability to spread through aerosols led to its classification as a potential biowarfare/bioterrorism agent. The closely related but opportunistic pathogenic species B. thailandensis has been widely used as a laboratory infection model to study melioidosis, as it shares an identical intracellular life cycle with B. mallei and B. pseudomallei. The bacteria invade phagocytic and nonphagocytic cells using T3SS (type III secretion system)-injected effector proteins and quickly escape from the endocytic compartment into the host cytosol (5, 6). Once cytosolic, B. thailandensis replicates and uses actin-based motility by coopting the host Arp2/3 complex through the bacterial protein BimA (6, 7), which allows it to form protrusions and spread to neighboring cells in a process requiring a type VI secretion system (T6SS). A hallmark of *Burkholderia* cell-to-cell spread is host cell fusion and the formation of so-called multinucleated giant cells (MNGCs). The presence of MNGCs has been observed in the tissues of patients with melioidosis (8), and they are thought to occur through T6SS effectors, specifically the T6SS protein VgrG5 (6, 9, 10).

Interferons (IFNs) are central cytokines in modulating host cell-autonomous defense and innate immune responses against a wide variety of pathogens. IFN signaling pathways induce the expression of IFN-stimulating genes (ISGs), which encode effector proteins that participate in immunity against viruses, bacteria, and protozoan parasites (11–15). Prominent among the ISGs are the guanylate-binding proteins (GBPs), a family of dynamin-like large GTPases with the ability to target intracellular parasites and Gram-negative bacteria, thus triggering inflammasome activation and antimicrobial mechanisms in both mouse and human cells (11–13). The role of GBPs in inflammasome activation is best studied for human GBP1, which has the ability to directly interact with lipopolysaccharide (LPS), the major component of the Gram-negative bacterial outer membrane. By functioning as a bona fide LPS sensor, human GBP1 (hGBP1) assembles a platform that recruits other GBP family members (hGBP2 to -4 [hGPB2-4]) (16) and caspase-4 directly on the surface of cytosolic Salmonella enterica serovar Typhimurium or Shigella flexneri cells (17–21). This in turn allows the activation of the so-called noncanonical inflammasome via LPS-induced caspase-4 oligomerization and activation, which cleaves the pore-forming cell death effector gasdermin D (GSDMD) to induce proinflammatory pyroptosis and interleukin-18 (IL-18) release (22, 23). In mouse macrophages, however, additional IFN-inducible GTPases such as immunity-related GTPases (IRGs) (which are not present in human cells except for an IRGM truncated form and immunity-related GTPases (IRGs) [24]) seem to play an antimicrobial role (25–28). It has been shown that mouse GBPs recruit Irgb10 onto bacterial surfaces to induce bacteriolysis and the release of bacterial pathogen-associated molecular patterns (PAMPs) that induce inflammasome activation and pyroptosis (25, 27, 28).

In a mouse model of infection, IFNs have been proposed to play a protective role in restricting *Burkholderia* infection by inducing *Casp11* upregulation (29). IFN production during *Burkholderia* infection, however, seems to be only a second layer of defense and occurs as a consequence of *Burkholderia*-induced activation of the canonical NLRC4 inflammasome and subsequent IL-18 secretion (30–32). Recent work has also suggested that in mouse macrophages, GBP coating of *Burkholderia* cells restricts cell fusion by preventing bacterial actin-based motility and spread (33). Unlike the mouse infection model, where data clearly support a role for IFNs in mediating *Burkholderia* restriction, little is known about the role of this cytokine in human cells, specifically in epithelial cells, in response to B. thailandensis.

Here, we provide evidence that in human epithelial cells, GBP1 restricts B. thailandensis-induced MNGC formation and that GBP1-dependent restriction is mediated by caspase-4-induced pyroptosis of infected cells and independent of restricting bacterial motility. Moreover, we show that the IFN-mediated restriction of B. thailandensis expands to several physiologically more relevant human cell lines, such as keratinocytes (HaCaT) and bronchial epithelial cells (HBEC3-KT). Finally, we observe that even in primary human macrophages, IFN priming confers a much more efficient clearance of *Burkholderia* infections.

## RESULTS

### Interferons restrict MNGC formation in epithelial cells during B. thailandensis infection.

**FIG 1:**
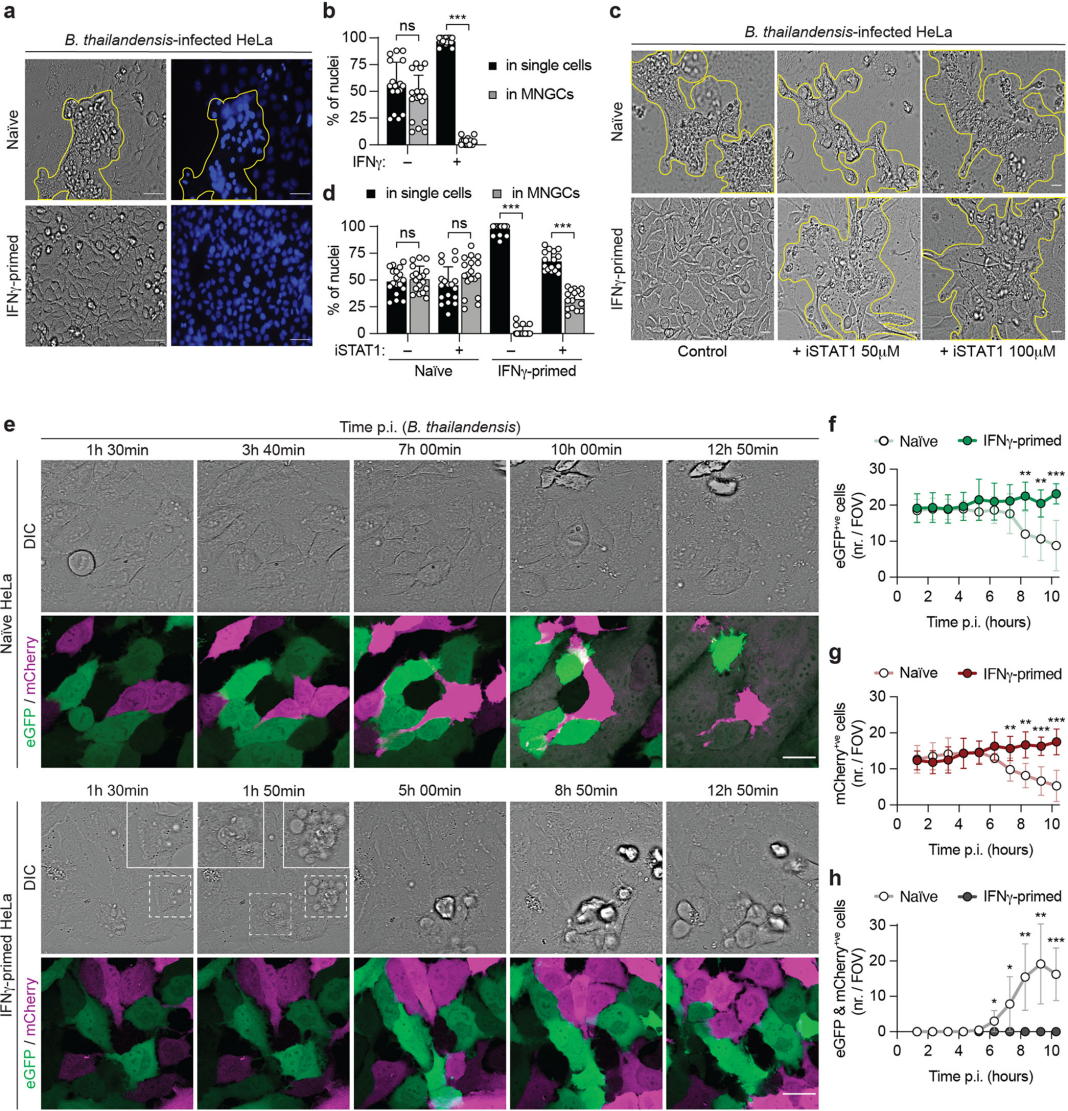
Interferons restrict multinucleated giant cell (MNGC) formation in epithelial cells during B. thailandensis infection. (a) Representative phase-contrast images (magnification, ×40) of naive or IFN-γ-primed HeLa cells 20 h after infection with B. thailandensis (MOI of 0.3). The corresponding DNA was stained with Hoechst stain (right), and clustered nuclei, indicating MNGCs, are highlighted by yellow outlines. Bars, 100 μm. (b) Percentage of nuclei found in MNGCs or in single cells, determined by counting nuclei of naive or IFN-γ-primed HeLa cells 20 h after infection with B. thailandensis (MOI of 0.3) in 6 fields of view under each experimental condition. (c) Representative phase-contrast images (magnification, ×40) of naive or IFN-γ-primed HeLa cells infected with B. thailandensis (MOI of 100) in the presence or absence of the STAT1 inhibitor fludarabine at the indicated concentrations. Bars, 100 μm. Yellow outlines correspond to clusters of cells forming MNGCs. (d) Percentage of nuclei in MNGCs or in single cells, determined by counting nuclei of naive or IFN-γ-primed HeLa cells 20 h after infection with B. thailandensis (MOI of 0.3) in the presence or absence of the STAT1 inhibitor (100 μM) in 6 fields of view under each experimental condition. (e) Time-lapse fluorescence confocal microscopy of naive or IFN-γ-primed HeLa cells stably expressing Dox-inducible eGFP or mCherry. eGFP and mCherry expression was induced with 1 μg/ml of Dox 4 h prior to infection. Cells were cocultured at a 1:1 ratio and infected with B. thailandensis (MOI of 50). Images were acquired every 10 min. Bars, 30 μm. DIC, differential interference contrast. (f to h) Number of eGFP-positive (f), mCherry-positive (g), or eGFP/mCherry-double-positive (h) cells per field of view (FOV) in naive or IFN-γ-primed HeLa cells infected with B. thailandensis. Data are representative of results from at least three independent experiments performed in triplicate (a, c, and e). Graphs show the means ± standard deviations (SD), and data are pooled from two (d) or three (b) independent experiments performed in duplicates or six independent movies (f to h). ns, not significant; *, *P < *0.05; **, *P < *0.01; ***, *P < *0.001 (by 2-way analysis of variance [ANOVA] [b and d] or a parametric *t* test [f to h]).

In a mouse model of infection, type I and type II IFNs have been described to participate in the immune response against B. thailandensis (29, 33, 34). However, it has been suggested that they play differential roles in epithelial cells and macrophages (35). Moreover, how IFNs regulate defense against this bacterium in the human system remains poorly understood. To gain more insights into the role of IFN priming in protecting human epithelial cells against B. thailandensis infection, we infected HeLa cells with B. thailandensis and monitored the formation of MNGCs, a hallmark of B. thailandensis spread and replication. Microscopy-based analysis showed that by 20 h postinfection (p.i.), naive HeLa cells formed large cell clusters with tightly packed nuclei (Fig. 1a; see also Fig. S1a in the supplemental material), consistent with the formation of cell aggregates known as MNGCs (6, 36). Through image-based quantification, we estimated that approximately 50% of all nuclei belonged to giant cells (Fig. 1b). Strikingly, B. thailandensis-induced MNGC formation was almost completely restricted in IFN-γ-primed HeLa cells (Fig. 1a and b and Fig. S1a). Importantly, IFN-γ did not interfere with bacterial uptake as assayed by CFU counting (Fig. S1b).

10.1128/mBio.02054-21.1 FIG S1 (a) Representative phase-contrast images (magnification, ×40) of naive or IFN-γ-primed HeLa cells 20 h after infection with B. thailandensis (MOI of 100). Bars, 100 μm. (b) Assessment of bacterial invasion in naive or IFN-γ-primed HeLa cells infected with B. thailandensis (MOI of 100). (c) Immunoblots for GBP1 and tubulin (loading control) in cell lysates of naive or IFN-γ-primed HeLa cells 20 h after infection with B. thailandensis (MOI of 100) in the presence or absence of the STAT1 inhibitor. (d) Schematic representation of the coculture model of naive and IFN-γ-primed HeLa cells expressing Dox-inducible eGFP (green) or mCherry (purple) employed to track multinucleation events in response to B. thailandensis infection. (e) Time-lapse fluorescence confocal microscopy of naive or IFN-γ-primed HeLa cells stably expressing Dox-inducible eGFP or mCherry. Cells were cocultured at a 1:1 ratio and infected with B. thailandensis (MOI of 50). Images were acquired every 10 min. Bars, 30 μm. (f) Immunoblotting for GBP1, GBP2, caspase-4, and tubulin (loading control) in the cell lysate of naive or IFN-γ-primed wild-type and *GBP1*^–/–^ HeLa cells. (g) Representative phase-contrast images (magnification, ×40) of naive or IFN-γ-primed HeLa cells 20 h after infection with B. thailandensis (MOI of 100). Bars, 100 μm. (h) Assessment of bacterial invasion in naive or IFN-γ-primed wild-type and *GBP1*^–/–^ HeLa cells infected with B. thailandensis (MOI of 100). Data are representative of results from two (c and f) or at least three (a, e, and g) independent experiments performed in triplicate. Graphs show the means ± SD, and data are pooled from three independent experiments performed in triplicates (b and h). ns, not significant (by a parametric *t* test [b] or 2-way ANOVA [h]). Download FIG S1, TIF file, 1.7 MB. Copyright © 2021 Dilucca et al. 2021 Dilucca et al. https://creativecommons.org/licenses/by/4.0/ This content is distributed under the terms of the Creative Commons Attribution 4.0 International license.

Two main classes of IFNs have been described: type I, which includes many IFN types, such as IFN-α and IFN-β, and type II, e.g., IFN-γ. Following cognate receptor binding, both classes trigger a downstream signaling pathway through STAT1 that culminates in the transcription of interferon-stimulated genes (ISGs) (37). In order to confirm the role of IFN signaling in restricting MNGC formation, we used the STAT1 inhibitor fludarabine. IFN-γ-primed HeLa cells pretreated with fludarabine lost the ability to restrict MNGC formation upon B. thailandensis infection, almost to the levels found in naive cells (Fig. 1c and d). Immunoblotting confirmed the inhibition of the IFN signaling pathway, as the expression of hGBP1 (an ISG product selected as a marker to assess STAT1 inhibition) was partially reduced by fludarabine (Fig. S1c).

To better understand the cell-cell fusion dynamics and further confirm the IFN-γ-dependent restriction of MNGC formation during B. thailandensis infection, we used a coculture model of HeLa cells expressing doxycycline (Dox)-inducible enhanced green fluorescent protein (eGFP) (HeLa-eGFP) or Dox-inducible mCherry (HeLa-mCherry). We then performed time-lapse fluorescence confocal microscopy to track cell fusion and MNGC formation, which is characterized by the mixing and colocalization of both cytosolic fluorescent proteins (Fig. S1d). We found that infected naive cells started to fuse at about 6 to 8 h p.i. (Fig. 1e, top; Fig. S1e; and Movie S1), resulting in decreases in eGFP- or mCherry-positive cells (Fig. 1f and g) and concomitant increases in eGFP/mCherry-double-positive cells (Fig. 1h). Eventually, MNGCs were formed as a result of the fusion of several cells (Fig. 1e, top; Fig. S1e; and Movie S1). In contrast, no cell-cell fusion and MNGCs were detected in infected IFN-γ-primed cells (Fig. 1e, bottom; Fig. 1f to h; Fig. S1e; and Movie S2).

10.1128/mBio.02054-21.4 MOVIE S1 Naive HeLa cells infected with B. thailandensis undergo cell-to-cell fusion, forming multinucleated giant cells. Time-lapse fluorescence confocal microscopy of naive HeLa cells stably expressing eGFP (green) or mCherry (purple) and infected with B. thailandensis are shown. Images were acquired every 10 min. Bar, 30 μm. Download Movie S1, AVI file, 9.9 MB. Copyright © 2021 Dilucca et al. 2021 Dilucca et al. https://creativecommons.org/licenses/by/4.0/ This content is distributed under the terms of the Creative Commons Attribution 4.0 International license.

10.1128/mBio.02054-21.5 MOVIE S2 IFN-γ-primed HeLa cells infected with B. thailandensis do not undergo cell-to-cell fusion. Time-lapse fluorescence confocal microscopy of IFN-γ-primed HeLa cells stably expressing eGFP (green) or mCherry (purple) and infected with B. thailandensis are shown. Images were acquired every 10 min. Bar, 30 μm. Download Movie S2, AVI file, 12.3 MB. Copyright © 2021 Dilucca et al. 2021 Dilucca et al. https://creativecommons.org/licenses/by/4.0/ This content is distributed under the terms of the Creative Commons Attribution 4.0 International license.

Altogether, these findings point out that in human epithelial cells, B. thailandensis spread and the resulting multinucleated cell formation are impaired in an IFN-dependent manner.

### Human GBPs restrict MNGC formation.

**FIG 2:**
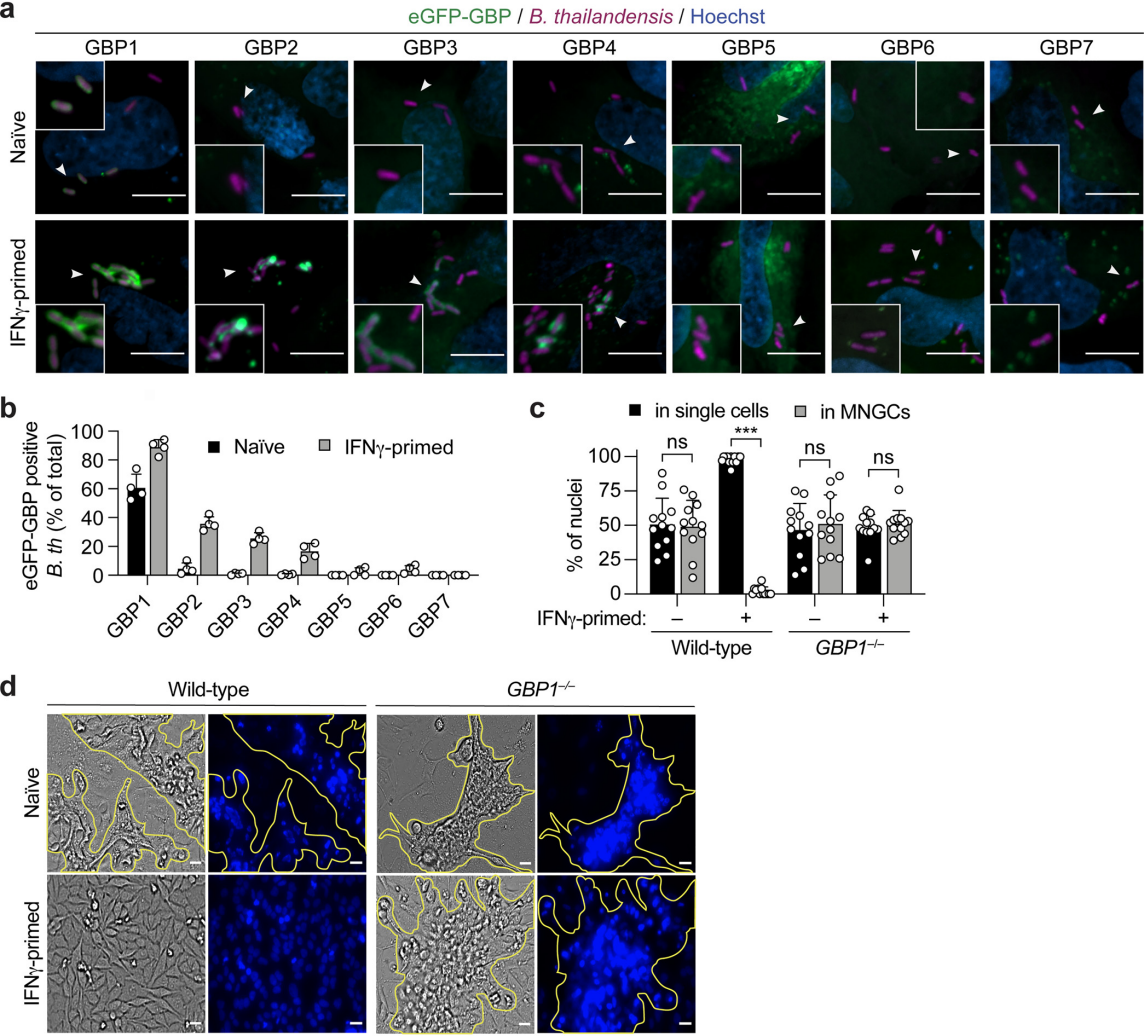
Human GBPs restrict MNGC formation. (a) Representative fluorescence confocal microscopy images of naive or IFN-γ-primed HeLa cells expressing N-terminally tagged eGFP–GBP1-7 and infected with B. thailandensis*-*mCherry (MOI of 30) for 3 h. DNA was stained by Hoechst stain. Bars, 10 μm. (b) Percentage of intracellular eGFP–GBP1-7-positive B. thailandensis (*B. th*) bacteria in naive or IFN-γ-primed HeLa cells at 3 h p.i. At least 200 to 300 bacteria per well were counted. (c) Percentage of nuclei in MNGCs or in single cells, determined by counting the nuclei of naive or IFN-γ-primed wild-type or *GBP1^–/–^* HeLa cells 20 h after infection with B. thailandensis (MOI of 0.3) in 6 fields of view under each experimental condition. (d) Representative phase-contrast images (magnification, ×40) of naive or IFN-γ-primed wild-type or *GBP1^–/–^* HeLa cells 20 h after infection with B. thailandensis (MOI of 0.3). The corresponding DNA was stained with Hoechst stain (right), and clustered nuclei, indicating MNGCs, are highlighted by yellow outlines. Bars, 100 μm. Graphs show the means ± SD, and data are pooled from two independent experiments performed in duplicate (b and c) or are representative of results from at least three independent experiments performed in triplicate (a and d). ***, *P < *0.001; ns, not significant (by 2-way ANOVA [c]).

GBPs are well-known ISGs and have been shown to be crucial for the proper activation of innate immune defense mechanisms against Gram-negative bacteria, protozoan parasites, and viruses (12, 14, 15, 38). It was recently proposed that during B. thailandensis infection of mouse bone marrow-derived macrophages (BMDMs), mouse GPBs (mGBPs) contribute to restricting actin-based bacterial spread and cell-cell fusion, thus also reducing bacterium-induced pathology *in vivo* (33). Although it has been reported that B. thailandensis is targeted by GBP1 in human epithelial cells (16), the role of GBPs during *Burkholderia* infection of human cells, and specifically epithelial cells, is largely unknown. We first assessed if intracellular B. thailandensis is targeted by GBPs in HeLa cells ectopically expressing N-terminally eGFP-tagged GBPs (eGFP-GBPs). In accordance with a previous study (16), we observed that B. thailandensis was targeted by eGFP-GBP1 in IFN-γ-primed cells, where around 90% of the intracellular bacteria are GBP1 coated at 3 h p.i. (Fig. 2a and b). In naive cells, GBP1 was also associated with a high percentage of intracellular B. thailandensis bacteria, suggesting that it also senses cytosolically exposed LPS on the surface of this bacterium, similar to its role in Salmonella or *Shigella* infections (17–21). On the other hand, GBP2, -3, and -4 coated 40 to 20% of intracellular *Burkholderia* bacteria only in IFN-γ-primed but not in naive HeLa cells, corroborating the notion that their recruitment to cytosolic Gram-negative bacteria is driven by additional effectors that act upstream, namely, GBP1 (16–21). Similar to what has been shown upon *Shigella* infection (16), a very low percentage of bacteria positive for eGFP-GBP5, -6, and -7 was detected in both naive and IFN-γ-primed HeLa cells (Fig. 2a and b), suggesting that these three GBPs do not play a major role in recognizing this pathogen.

In order to investigate the possible role of hGBPs in restricting MNGC formation upon IFN-γ priming, we used *GBP1*^–/–^ HeLa cells previously generated by CRISPR-Cas9 genome editing (17). We found that hGBP1 is involved in restricting MNGC formation upon B. thailandensis infection, as IFN-γ-primed *GBP1*-deficient HeLa cells formed MNGCs in a manner comparable to that of wild-type naive cells (Fig. 2c and d and Fig. S1f and g) without affecting bacterial entry into cells (Fig. S1h). Collectively, these data suggest that hGBPs are the IFN-dependent downstream effectors responsible for restricting MNGC formation and B. thailandensis spread.

### GBP1 promotes caspase-4-dependent pyroptosis and restricts MNGC formation and B. thailandensis replication.

Recent studies have shown that in human epithelial cells and macrophages, GBPs are required for noncanonical inflammasome activation by targeting LPS and assembling a caspase-4-activating platform on the surface of cytosolic Salmonella and *Shigella* bacteria (17–21). Polymerized GBP1 on the bacterial surface was also proposed to act as an LPS surfactant that increases bacterial susceptibility to antimicrobial effectors by destabilizing the bacterial outer membrane (21). Moreover, the GBP coat assembled on *Shigella* cells appears to have an additional function of inhibiting actin-based motility and consequent bacterial cell-to-cell spread (16, 39). This same mechanism has recently been proposed to prevent B. thailandensis invasion of neighboring cells in murine BMDMs (33). Therefore, we speculated that one or several of these mechanisms might be responsible for the GBP1-dependent restriction of MNGC formation in human epithelial cell lines in response to *Burkholderia* infection (Fig. S2a).

10.1128/mBio.02054-21.2 FIG S2 (a) Schematic representation of known GBP1-dependent mechanisms that could restrict bacterial spreading. (b) Time-lapse fluorescence confocal microscopy of IFN-γ-primed HeLa cells expressing N-terminally eGFP-tagged GBP1 (green) and infected with B. thailandensis*-*mCherry (MOI of 30) (purple). DIC, differential interference contrast. Images were acquired every 20 min. Bars, 10 μm. Arrows point to plasma membrane blebbing of a pyroptotic cell. Arrowheads point to bacteria that were targeted by GBP1. (c) Percentage of IFN-γ-primed HeLa cells in which GBP1 was recruited to B. thailandensis*-*mCherry and that underwent pyroptosis or no pyroptosis. Cells expressing eGFP-GBP1 were infected as described above for panel b, and time-lapse confocal microscopy was performed. A total of 15 infected cells from 3 independent experiments were analyzed. (d) Percentage of PI uptake in naive or IFN-γ-primed HeLa cells infected with B. thailandensis (MOI of 200). (e) Time-lapse fluorescence microscopy images of IFN-γ-primed HeLa cells expressing eGFP–caspase-4 (green) and infected with B. thailandensis*-*mCherry (MOI of 30) (purple). Images were acquired every 8 min. Bar, 10 μm. Arrows point to plasma membrane blebbing of a pyroptotic cell. Arrowheads point to bacteria that were targeted by caspase-4. (f) Percentage of IFN-γ-primed HeLa cells in which caspase-4 was recruited to B. thailandensis*-*mCherry and that underwent pyroptosis or no pyroptosis. Cells expressing caspase-4–eGFP were infected as described above for panel e, and time-lapse confocal microscopy was performed. A total of 20 infected cells from 4 independent experiments were analyzed. (g) Percentage of PI uptake in wild-type, *CASP4*^–/–^, *GSDMD*^–/–^, and *GBP1*^–/–^ IFN-γ-primed HeLa cells infected with B. thailandensis (MOI of 200). (h) Representative phase-contrast images (magnification, ×40) of naive or IFN-γ-primed wild-type, *CASP4*^–/–^, *GSDMD*^–/–^, and *GBP1*^–/–^ HeLa cells 20 h after infection with B. thailandensis (MOI of 100). Bars, 100 μm. (i) Assessment of bacterial invasion in naive or IFN-γ-primed wild-type, *CASP4^–/–^*, *GSDMD^–/–^*, and *GBP1^–/–^* HeLa cells infected with B. thailandensis (MOI of 100). Data are representative of results from at least three independent experiments performed in triplicate (b, e, and h). Graphs show the means ± SD, and data are pooled from three independent experiments performed in triplicates (i) or are representative of results from two independent experiments performed in triplicates (d and g). In panels c and g, the area under the curve (AUC) under each experimental condition was calculated and analyzed by a parametric *t* test (d) or one-way ANOVA (g). *, *P < *0.05; **, *P < *0.01; ns, not significant (by 2-way ANOVA [i]). Download FIG S2, TIF file, 1.5 MB. Copyright © 2021 Dilucca et al. 2021 Dilucca et al. https://creativecommons.org/licenses/by/4.0/ This content is distributed under the terms of the Creative Commons Attribution 4.0 International license.

**FIG 3:**
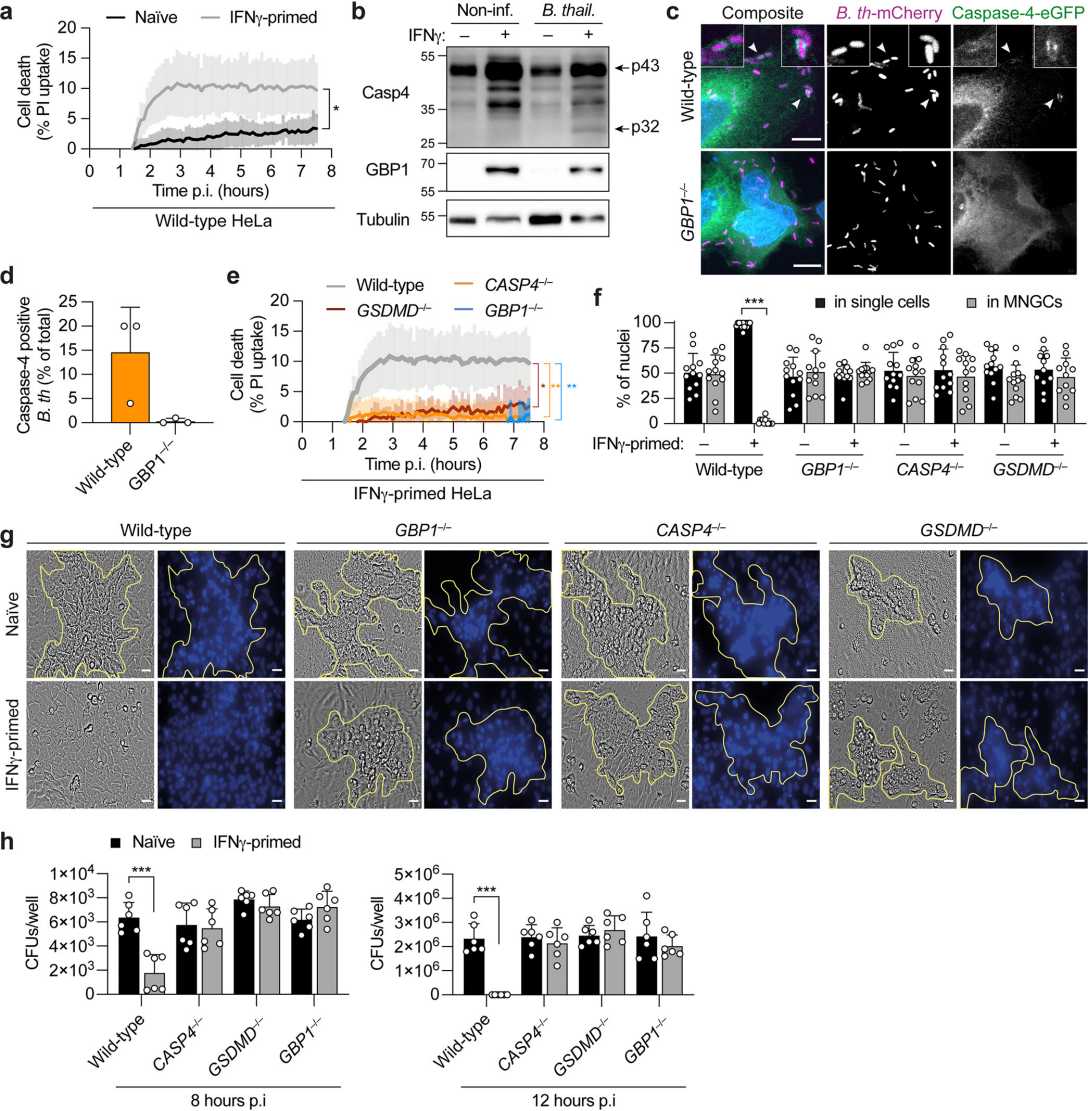
GBP1 promotes caspase-4-dependent pyroptosis and restricts multinucleated giant cell (MNGC) formation and B. thailandensis replication. (a) Percentage of PI uptake in naive or IFN-γ-primed HeLa cells infected with B. thailandensis (MOI of 200). (b) Immunoblotting for caspase-4 processing, GBP1, and tubulin (loading control) of combined supernatants and cell lysates of naive or IFN-γ-primed HeLa cells 6 h after infection with B. thailandensis (MOI of 200). (c) Representative fluorescence confocal microscopy images of IFN-γ-primed wild-type and *GBP1^–/–^* HeLa cells treated with z-VAD-FMK (carbobenzoxy-valyl-alanyl-aspartyl-[*O*-methyl]-fluoromethylketone) (10 μM). Cells expressing caspase-4–eGFP were infected with B. thailandensis*-*mCherry (MOI of 100) for 6 h. DNA was stained by Hoechst stain. Bars, 10 μm. (d) Percentage of intracellular caspase-4–eGFP-positive B. thailandensis-mCherry bacteria in IFN-γ-primed HeLa cells treated with z-VAD-FMK (10 μM) at 6 h p.i. At least 100 to 200 bacteria per well were counted. (e) Percentage of PI uptake in wild-type, *GBP1^–/–^*, *CASP4^–/–^*, and *GSDMD^–/–^* IFN-γ-primed HeLa cells infected with B. thailandensis (MOI of 200). (f) Percentage of nuclei in MNGCs or in single cells, determined by counting the nuclei of naive or IFN-γ-primed wild-type, *GBP1^–/–^*, *CASP4^–/–^*, and *GSDMD^–/–^* HeLa cells 20 h after infection with B. thailandensis (MOI of 0.3) in 6 fields of view under each experimental condition. (g) Representative phase-contrast images (magnification, ×40) of naive or IFN-γ-primed wild-type, *GBP1^–/–^*, *CASP4^–/–^*, and *GSDMD^–/–^* HeLa cells 20 h after infection with B. thailandensis (MOI of 0.3). The corresponding DNA was stained with Hoechst stain (right), and clustered nuclei, indicating MNGCs, are highlighted by yellow outlines. Bars, 100 μm. (h) Assessment of bacterial invasion in naive or IFN-γ-primed wild-type, *GBP1^–/–^*, *CASP4^–/–^*, and *GSDMD^–/–^* HeLa cells 8 or 12 h after infection with B. thailandensis (MOI of 100). Data are representative of results from at least three independent experiments (c, d, and g) or two independent experiments (b). Graphs show the means ± SD, and data are pooled from two independent experiments performed in duplicate (f) or triplicate (h) or are representative of results from at least two independent experiments performed in triplicate (a and e). For panels a and e, the area under the curve (AUC) under each experimental condition was calculated, and data were analyzed by a parametric *t* test (a) or one-way ANOVA (e). *, *P < *0.05; **, *P < *0.01; ***, *P < *0.001 (by 2-way ANOVA [f and h]).

Upon B. thailandensis infection, IFN-γ-primed HeLa cells showed signs of cell ballooning and blebbing, which are hallmarks of pyroptosis (Fig. 1e). Furthermore, a closer analysis of time-lapse confocal microscopy images of B. thailandensis-infected IFN-γ-primed HeLa cells showed that GBP1 targeting to cytosolic bacteria was followed by pyroptotic cell death in the majority of cases (as observed by nuclear condensation and plasma membrane swelling), with a concomitant restriction of bacterial replication (Movie S3 and Fig. S2b and c). The activation of the noncanonical inflammasome leads to caspase-4 activation and autoprocessing and the subsequent cleavage of the pyroptotic executor GSDMD (40). The cleaved GSDMD N-terminal domain forms pores that result in propidium iodide (PI) uptake, which can be used as a marker of lytic cell death. In the early stages of B. thailandensis infection, IFN-γ-primed wild-type HeLa cells showed a higher percentage of PI-positive cells than did naive cells (Fig. 3a and Fig. S2d). Furthermore, we observed that B. thailandensis infection resulted in caspase-4 activation only in IFN-γ-primed cells (Fig. 3b, p32 fragment), which is in accordance with what has been observed during infection of epithelial cells with other cytosolic Gram-negative bacteria (17, 18). According to recent studies, coating of the surface of cytosolic Gram-negative bacteria by GBPs facilitates caspase-4 recruitment and activation, initiating the downstream pathway that culminates in the lytic death of the infected cell (17–21). Caspase-4 localization during B. thailandensis infection of IFN-γ-primed HeLa cells was assessed by fluorescence confocal microscopy. As expected, caspase-4–eGFP was found to be recruited to cytosolic B. thailandensis in a GBP1-dependent manner (Fig. 3c and d, Fig. S3e, and Movie S4) but not to the same levels as GBP1 targeting (Fig. 2a and b). Furthermore, the recruitment of caspase-4 to bacteria correlated with pyroptosis of the infected cell, as determined by the appearance of the typical pyroptotic morphology (Fig. S2e and f and Movie S4). To test if this cell death was caused by noncanonical inflammasome activation, we used wild-type, *CASP4*^–/–^, *GSDMD*^–/–^, and *GBP1*^–/–^ HeLa cells, which have been previously generated and verified (17). We observed that caspase-4, GSDMD, and GBP1 were required for PI influx, indicating that the cell lysis observed upon B. thailandensis infection was triggered by noncanonical inflammasome activation (Fig. 3e and Fig. S2g).

10.1128/mBio.02054-21.3 FIG S3 (a) Representative confocal microscopy images of naive and IFN-γ-primed *CASP4^–/–^* HeLa cells infected with B. thailandensis*-*mCherry (purple) for 5 h (MOI of 30). F-actin (green) was labeled with CellMask green actin tracking stain. Bars, 10 μm. (b) Percentage of Shigella flexneri bacteria with actin tails in naive and IFN-γ-primed wild-type and *GBP1*^–/–^ HeLa cells at 2 h p.i. (MOI of 30). At least 100 to 200 bacteria per coverslip were counted. (c) Representative phase-contrast images (magnification, ×40) of naive or IFN-γ-primed HBEC3-KT and HaCaT cells 20 h after infection with B. thailandensis (MOI of 100). Bars, 100 μm. (d and e) Assessment of bacterial invasion in naive or IFN-γ-primed HBEC3-KT cells (d) and HaCaT cells (e) infected with B. thailandensis (MOI of 100). (f and g) Immunoblots for CASP4, GSDMD, GBP1, and tubulin (loading control) in cell lysates from IFN-γ-primed HBEC3-KT (f) or HaCaT (g) cells 24 h after transfection with nontargeting siRNA (NT) or siRNA against CASP4, GSDMD, and GBP1. (h) Representative phase-contrast images (magnification, ×40) of naive or IFN-γ-primed hMDMs 20 h after infection with B. thailandensis (MOI of 100). Bars, 100 μm. (i) Immunoblots for CASP4, GBP1, and tubulin (loading control) in cell lysates from naive and IFN-γ-primed hMDMs. (l) Schematic representation of caspase-4-mediated noncanonical activation of NLRP3. Data are representative of results from at least two (f to i), three (c), or five (a) independent experiments. Graphs show the means ± SD, and data are pooled from two independent experiments performed in duplicates (b) or triplicates (d and e). ***, *P < *0.001; ns, not significant (by a parametric *t* test [b, d, and e]). Download FIG S3, TIF file, 0.9 MB. Copyright © 2021 Dilucca et al. 2021 Dilucca et al. https://creativecommons.org/licenses/by/4.0/ This content is distributed under the terms of the Creative Commons Attribution 4.0 International license.

10.1128/mBio.02054-21.6 MOVIE S3 GBP1 recruitment to cytosolic B. thailandensis is followed by pyroptosis. Time-lapse fluorescence confocal microscopy of IFN-γ-primed HeLa cells expressing eGFP-GBP1 (green) and infected with B. thailandensis-mCherry (purple) is shown. (Left) DIC shows a cell undergoing pyroptosis, as seen by plasma membrane ballooning and nuclear condensation. (Right) Composite image. Bar, 10 μm. Download Movie S3, AVI file, 2.0 MB. Copyright © 2021 Dilucca et al. 2021 Dilucca et al. https://creativecommons.org/licenses/by/4.0/ This content is distributed under the terms of the Creative Commons Attribution 4.0 International license.

10.1128/mBio.02054-21.7 MOVIE S4 Caspase-4 recruitment to cytosolic B. thailandensis is followed by pyroptosis. Time-lapse fluorescence confocal microscopy of IFN-γ-primed HeLa cells expressing caspase-4–eGFP (green) and infected with B. thailandensis-mCherry (purple) is shown. (Left) DIC shows a cell undergoing pyroptosis, as seen by plasma membrane ballooning and nuclear condensation. (Right) Composite image. Bar, 10 μm. Download Movie S4, AVI file, 8.8 MB. Copyright © 2021 Dilucca et al. 2021 Dilucca et al. https://creativecommons.org/licenses/by/4.0/ This content is distributed under the terms of the Creative Commons Attribution 4.0 International license.

Quantification of the multinucleation events confirmed that wild-type naive HeLa and IFN-γ-primed *CASP4*^–/–^, *GSDMD*^–/–^, and *GBP1*^–/–^ cells have similar percentages of nuclei associated with MNGCs (Fig. 3f). Furthermore, IFN-γ-primed *CASP4*^–/–^, *GSDMD*^–/–^, and *GBP1*^–/–^ HeLa cells infected with B. thailandensis formed MNGCs in a similar manner and with comparable size to those observed in wild-type naive cells (Fig. 3f and g and Fig. S3h). Notably, IFN-γ priming restricted intracellular B. thailandensis replication in a caspase-4-, GSDMD-, and GBP1-dependent manner at 8 and 12 h p.i. (Fig. 3h), without affecting bacterial entry (Fig. S3i).

Together with the observation that the IFN-dependent expression of GBPs is important for preventing MNGC formation (Fig. 2c and d), these data show that GBP1 induces rapid death of the infected cells by triggering caspase-4-dependent pyroptosis, thus restricting B. thailandensis-induced cell-to-cell fusion and bacterial spread and replication.

### GBP1 does not impair actin-based motility or promote direct bacteriolysis of cytosolic B. thailandensis.

**FIG 4:**
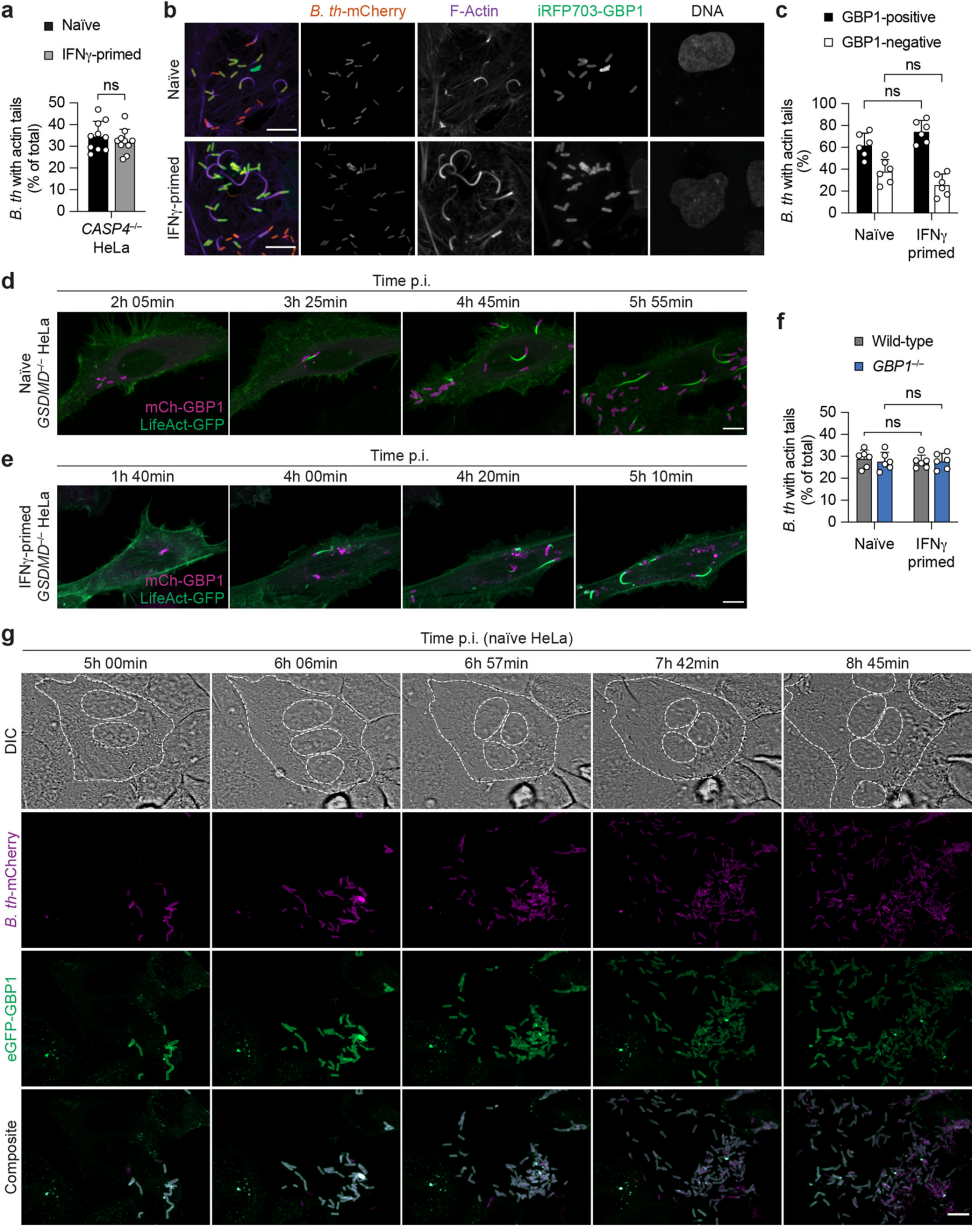
GBP1 does not impair actin-based motility or promote direct bacteriolysis of cytosolic B. thailandensis. (a) Percentage of B. thailandensis bacteria with actin tails at 5 h p.i. in naive and IFN-γ-primed *CASP4^–/–^* HeLa cells (MOI of 30). At least 100 to 200 bacteria per coverslip were counted. (b) Representative fluorescence confocal microscopy images of naive and IFN-γ-primed *CASP4^–/–^* HeLa cells expressing iRFP703-GBP1 and infected with B. thailandensis*-*mCherry (MOI of 30) for 5 h. DNA was stained by Hoechst stain, and F-actin was labeled with CellMask green actin tracking stain. Bars, 10 μm. (c) Percentage of B. thailandensis bacteria with actin tails that are GBP1 positive or negative in naive and IFN-γ-primed *CASP4^–/–^* HeLa cells expressing iRFP703-GBP1. Cells were infected with B. thailandensis-mCherry for 5 h (MOI of 30) and fixed, and F-actin was labeled with CellMask green actin tracking stain. Between 100 and 300 bacteria were counted per coverslip. (d and e) Time-lapse fluorescence confocal microscopy images of naive (d) and IFN-γ-primed (e) *GSDMD^–/–^* HeLa cells expressing N-terminally mCherry-tagged GBP1 and LifeAct-eGFP infected with B. thailandensis (MOI of 50). Images were acquired every 5 min. Bars, 10 μm. (f) Percentage of B. thailandensis bacteria with actin tails in naive and IFN-γ-primed wild-type and *GBP1^–/–^* HeLa cells. Cells were treated with z-VAD-FMK (10 μM) and infected for 5 h at an MOI of 30. Between 100 and 300 bacteria were counted per coverslip. (g) Time-lapse fluorescence confocal microscopy of naive HeLa cells expressing N-terminally eGFP-tagged GBP1 and infected with B. thailandensis*-*mCherry (MOI of 50). MNGCs are indicated by dashed white lines. DIC, differential interference contrast. Images were acquired every 3 min. Bars, 10 μm. Data are representative of results from at least three independent experiments (b, d, e, and g). Graphs show the means ± SD, and data are pooled from three (c and f) or five (a) independent experiments performed in duplicate. ns, not significant (by a parametric *t* test).

After demonstrating that GBP1 triggers noncanonical inflammasome activation upon sensing cytosolic B. thailandensis in human epithelial cells, we also tested additional antimicrobial GBP-induced mechanisms that have been proposed previously (16, 33, 39, 41) (Fig. S2a). Cytosolic *Burkholderia* spp. are known to coopt the host actin polymerization machinery in order to spread from cell to cell (7). We first evaluated if IFN-γ priming and GBP1 affect actin tail polymerization on intracellular B. thailandensis cells by confocal microscopy. For this, we used *CASP4*-deficient HeLa cells to avoid IFN-γ-induced noncanonical activation and cell death. Surprisingly, we found that IFN-γ priming did not affect actin tail formation on intracellular B. thailandensis bacteria (Fig. 4a and Fig. S3a), contrary to the IFN-γ- and GBP1-dependent restriction of Shigella flexneri actin tail formation (39) (Fig. S3b). To test if GBP1-positive B. thailandensis cells were able to form actin tails, we infected naive or IFN-γ-primed *CASP4^–/–^* HeLa cells expressing iRFP703-GBP1 with B. thailandensis-mCherry for 5 h. Confocal microscopy analysis showed that in IFN-γ-primed cells, GBP1-positive bacteria formed actin tails to the same extent as in naive cells (Fig. 4b and c). The same was observed when we performed time-lapse fluorescence microscopy on LifeAct-GFP-expressing *GSDMD*^–/–^ infected HeLa cells; i.e., GBP1 coating of cytosolic B. thailandensis did not inhibit comet tail formation and actin-based bacterial motility in both naive and IFN-γ-primed cells (Fig. 4d and e and Movies S5 and S6). This suggests that GBP1, directly or in combination with other GBPs that oligomerize on the surface of cytosolic B. thailandensis, cannot impair B. thailandensis cell-to-cell spread via actin tails. To confirm this, we used *GBP1*-deficient HeLa cells and found that in infected wild-type cells, B. thailandensis formed actin tails to the same extent as in *GBP1*^–/–^ cells, both with and without IFN-γ priming (Fig. 4f). Interestingly, this is in contrast to what is observed in the case of Shigella flexneri infection, where GBP1 partially blocks actin tail formation (39) (Fig. S3b).

10.1128/mBio.02054-21.8 MOVIE S5 GBP1 recruitment to cytosolic B. thailandensis does not impair actin-based motility in naive HeLa cells. Time-lapse fluorescence confocal microscopy of naive *GSDMD^–/–^* HeLa cells expressing mCherry-GBP1 (purple) and LifeAct-eGFP (green) and infected with B. thailandensis is shown. Bar, 10 μm. Download Movie S5, AVI file, 2.8 MB. Copyright © 2021 Dilucca et al. 2021 Dilucca et al. https://creativecommons.org/licenses/by/4.0/ This content is distributed under the terms of the Creative Commons Attribution 4.0 International license.

10.1128/mBio.02054-21.9 MOVIE S6 GBP1 recruitment to cytosolic B. thailandensis does not impair actin-based motility in IFN-γ-primed HeLa cells. Time-lapse fluorescence confocal microscopy of IFN-γ-primed *GSDMD^–/–^* HeLa cells expressing mCherry-GBP1 (purple) and LifeAct-eGFP (green) and infected with B. thailandensis is shown. Bar, 10 μm. Download Movie S6, AVI file, 3.1 MB. Copyright © 2021 Dilucca et al. 2021 Dilucca et al. https://creativecommons.org/licenses/by/4.0/ This content is distributed under the terms of the Creative Commons Attribution 4.0 International license.

Furthermore, in naive HeLa cells infected with B. thailandensis-mCherry, the rapid oligomerization of GBP1 on the bacterial surface did not prevent bacterial replication in the host cytosol (Fig. 4g and Movie S7), and cell fusion and the formation of MNGCs were still observed (Fig. 4g, DIC [differential interference contrast]). This shows that GBP1 by itself does not appear to display antimicrobial activity when recruited to the bacteria in cells despite the previous observation that *in vitro*, the direct binding of GBP1 alone to bacteria disrupts cell envelope functions (21).

10.1128/mBio.02054-21.10 MOVIE S7 GBP1 recruitment to cytosolic B. thailandensis does not impair bacterial replication. Time-lapse fluorescence confocal microscopy of naive HeLa cells expressing eGFP-GBP1 (green) and infected with B. thailandensis*-*mCherry (purple) is shown. (Right) Composite image. Bar, 10 μm. Download Movie S7, AVI file, 11.5 MB. Copyright © 2021 Dilucca et al. 2021 Dilucca et al. https://creativecommons.org/licenses/by/4.0/ This content is distributed under the terms of the Creative Commons Attribution 4.0 International license.

Taken together, we conclude that GBP1-dependent restriction of MNGC formation in HeLa cells is not due to bacteriolysis or impaired bacterial actin-based motility within the host cytosol.

### Interferon has a protective role against B. thailandensis infection in human bronchial epithelial cells, keratinocytes, and primary macrophages.

**FIG 5:**
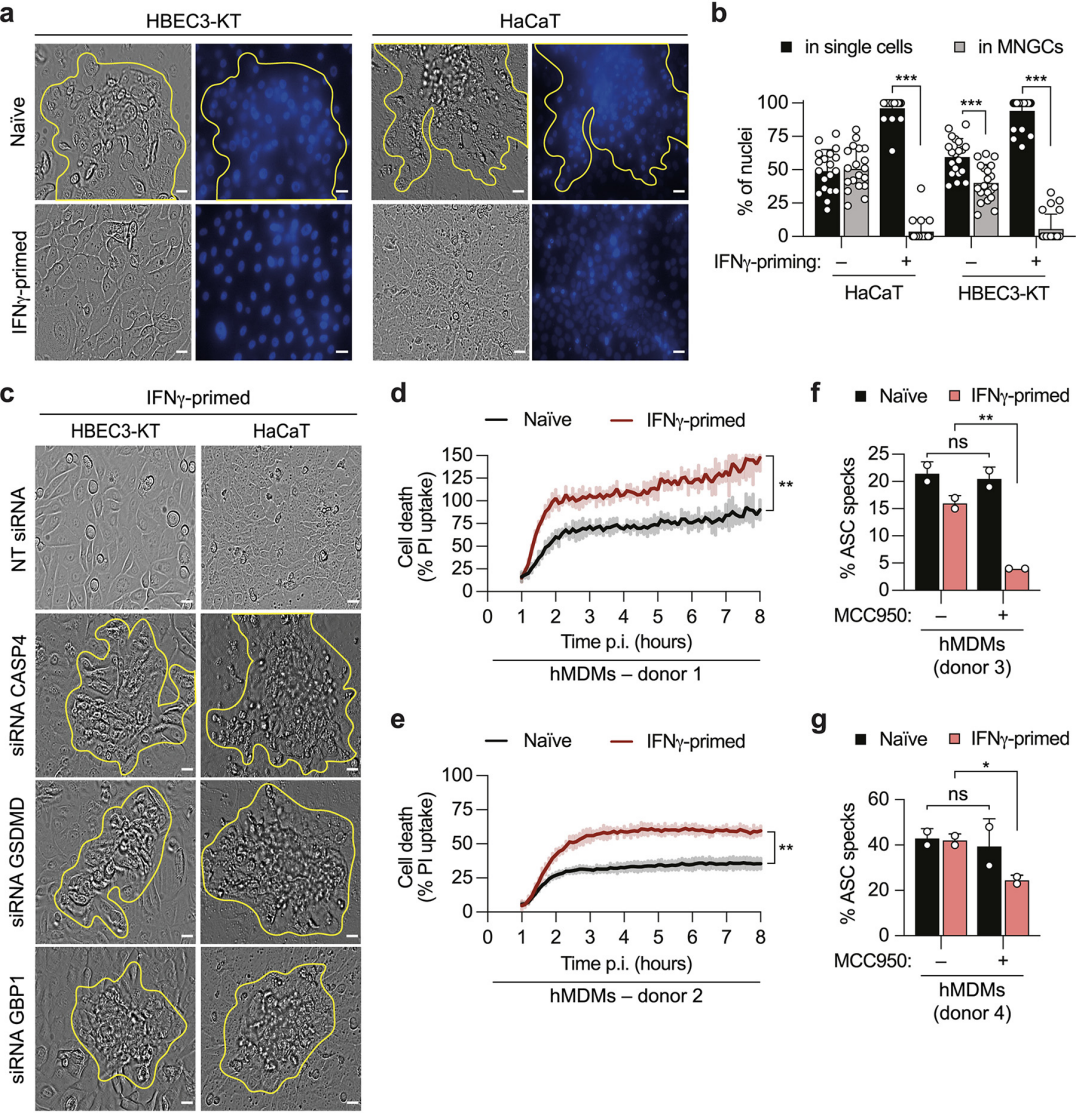
Interferon has a protective role against B. thailandensis infection in human bronchial epithelial cells, keratinocytes, and primary macrophages. (a and c) Representative phase-contrast images (magnification, ×40) of naive or IFN-γ-primed HBEC3-KT and HaCaT cells 20 h after infection with B. thailandensis (MOI of 100). In panel c, cells were pretreated with nontargeting (NT) siRNA or siRNA targeting CASP4, GSDMD, and GBP1, 24 h prior to infection. The corresponding DNA was stained with Hoechst stain, and multinucleated giant cells (MNGCs) are indicated by yellow outlines. Bars, 100 μm. (b) Percentage of nuclei in MNGCs or in single cells determined by counting the nuclei of naive or IFN-γ-primed HBEC3-KT and HaCaT cells 20 h after infection with B. thailandensis (MOI of 100) in 6 fields of view under each experimental condition. (d and e) Percentage of PI uptake in naive or IFN-γ-primed hMDMs infected with B. thailandensis (MOI of 30). (f and g) Percentage of ASC specks in naive or IFN-γ-primed hMDMs infected with B. thailandensis (MOI of 30) for 5 h in the presence or absence of the inhibitor MCC950. Data are representative of results from at least three independent experiments (a and c). Graphs show the means ± SD, and data are pooled from two independent experiments performed in triplicate (b) or are representative of results from two independent experiments performed in duplicate (f and g) or triplicate (d and e). The area under the curve (AUC) under each experimental condition was calculated (d and e), and data were analyzed by a parametric *t* test. *, *P < *0.05; **, *P < *0.01; ***, *P < *0.001; ns, not significant (by a parametric *t* test [f and g]).

Since bacteria of the *Burkholderia* genus employ different routes of infection (subcutaneous infection, inhalation, ingestion of contaminated particles, and aerosol) (1), we tested if IFN-γ priming restricts MNGC formation in human cell lines that are physiologically more relevant for *Burkholderia*-induced melioidosis, such as HBEC3-KT cells (human bronchial epithelial cells), HaCaT cells (human keratinocytes), and human primary monocyte-derived macrophages (hMDMs). Similar to HeLa cells (Fig. 1a and b), naive HBEC3-KT and HaCaT cells formed MNGCs after B. thailandensis infection, which was almost completely blocked upon IFN-γ priming (Fig. 5a and b and Fig. S3c). IFN-γ priming did not reduce bacterial uptake in these cells (Fig. S3d and e). We next determined whether the IFN-γ-dependent restriction of giant cell formation was promoted by noncanonical inflammasome activation, as shown for HeLa cells (Fig. 3e). In accordance with what we observed in HeLa cells, small interfering RNA (siRNA)-mediated knockdown of *CASP4*, *GSDMD*, or *GBP1* in HBEC3-KT and HaCaT cells abrogated the IFN-γ-mediated restriction of MNGC formation (Fig. 5c and Fig. S3f and g).

*Burkholderia* can invade both phagocytic and nonphagocytic cells. Among phagocytic cells, mainly macrophages and neutrophils take part in the immune response against this pathogen. Briefly, in a murine model of infection, *Burkholderia* is initially detected by macrophages through the Naip/Nlrc4 inflammasome (32), and the consequent IL-18 release triggers the production of IFN-γ whereby in neutrophils and macrophages, caspase-11 is upregulated (32). The subsequent noncanonical inflammasome activation in both cell types represents the critical step at which the B. thailandensis intracellular niche is removed. Therefore, we evaluated the role of IFN-γ priming in hMDMs during B. thailandensis infection. Interestingly, while unprimed murine BMDMs form MNGCs upon B. thailandensis infection (33), we did not detect the formation of MNGCs in either naive or IFN-γ-primed hMDMs (Fig. S3h). Instead, we observed robust induction of host cell death under both conditions, although the percentage of cell death, assessed by PI influx, was significantly higher in IFN-γ-primed hMDMs than in naive hMDMs (Fig. 5d and e). These results implied that analogously to IFN-γ-primed HeLa cells, the induction of cell lysis prevents MNGC formation in hMDMs. This cell death can be driven by IFN-independent (most likely via the NLRC4–caspase-1 axis) or IFN-dependent mechanisms, yet IFN signaling promotes a faster and more efficient way to activate GSDMD-induced pyroptosis and clear the bacteria. The latter most likely depends on the GBP-induced activation of the noncanonical inflammasome, as GBP1 expression in hMDMs was observed only after IFN-γ priming, whereas caspase-4 was constitutively expressed (Fig. S3i). NLRP3 can be activated downstream of caspase-4-induced GSDMD activation and cell death, further amplifying pyroptotic cell death via ASC (apoptosis-associated speck-like protein containing a CARD) speck formation and caspase-1 (Fig. S3l). To corroborate the role of B. thailandensis-induced noncanonical inflammasome activation in hMDMs, we treated naive or IFN-γ-primed cells with MCC950, a known selective NLRP3 inhibitor (42, 43) (Fig. S3l), and quantified the percentage of ASC specks by fluorescence microscopy. Inhibition of NLRP3 activation reduced ASC speck formation only in IFN-γ-primed and not in unprimed hMDMs (Fig. 5f and g), indicating that the noncanonical inflammasome is activated only in IFN-γ-primed hMDMs and that unprimed cells induce inflammasome activation by canonical inflammasomes. We also hypothesize that MNGCs were not observed in hMDMs because pyroptotic cell death occurred too quickly in response to B. thailandensis infection, even in naive cells. In conclusion, we demonstrate that the IFN-γ-dependent signaling axis described for HeLa cells upon *Burkholderia* infection is also found in other human epithelial cells as well as in human primary macrophages.

## DISCUSSION

This study provides the first evidence that in human epithelial cells, GBP-dependent noncanonical inflammasome activation prevents B. thailandensis*-*induced MNGC formation in the early stages of infection. Our results suggest that GBP1 impairs B. thailandensis cell-to-cell spread by triggering caspase-4-dependent pyroptosis of infected cells.

Previous work in mouse models of infection reported that both the Naip/NLRC4 inflammasome and the caspase-11 noncanonical inflammasome participate in the immune response against *Burkholderia* (29, 32, 35). Interestingly, these reports showed that both inflammasomes are connected given that caspase-1 activation in macrophages mediates IL-18 release to drive IFN-γ-dependent caspase-11 activation in epithelial cells. In agreement with this, we show that in human epithelial cells, IFNs and caspase-4-dependent pyroptosis provide protection against *Burkholderia* infection, whereas pyroptosis in primary hMDMs is driven by IFN-dependent and -independent mechanisms. The latter observation correlates with data in murine BMDMs that suggest that both the canonical and noncanonical pathways can be activated upon *Burkholderia* infection (29–31). The importance of noncanonical inflammasome activation in response to *Burkholderia* is particularly evident in HeLa cells, which lack canonical inflammasome pathways, but even human bronchial epithelial cells and keratinocytes mainly activate the noncanonical inflammasome in response to *Burkholderia*, suggesting that also in the human system, canonical inflammasome activation is restricted to professional immune cells.

Our work provides further support for the notion that human GBP1 acts as a cytosolic pattern recognition receptor that binds LPS in order to activate caspase-4 and restrict bacterial replication (17–21). Since GBP1 recruitment alone was not sufficient to restrict *Burkholderia* replication, we propose that GBP1-dependent caspase-4 activation is linked to its ability to recruit GBP2-4 to bacteria, which might amplify its effects. Whether the GBP1-4 coat exerts a strong LPS surfactant effect in cells to disrupt bacterial membranes or whether the coat directly interacts with and activates caspase-4 will need to be addressed by additional studies. It is also conceivable that GSDMD pores amplify noncanonical inflammasome activation by disrupting bacterial envelopes, as a previous study showed that recombinant GSDMD reduces bacterial viability *in vitro* upon caspase-1 processing and that bacteria were more susceptible to microbicidal effectors when harvested from mouse wild-type macrophages rather than *Gsdmd^–/–^* macrophages, which suggests that GSDMD can directly kill bacteria (44).

Disruption of the bacterial membrane was proposed to be the main mechanism by which mouse GBPs promote inflammasome activation, as GBPs were found to induce bacteriolysis by recruiting Irgb10 (27), a member of the IRG family of GTPases that are found in mouse but not human cells. However, a more recent study by the same authors suggested that during B. thailandensis infection, mouse GBPs do not lyse bacteria or activate the inflammasome but rather restrict B. thailandensis infection by inhibiting bacterial actin-based motility (33). Specifically, the higher number of multinucleation events observed in *Gbp2*^–/–^, *Gbp5*^–/–^, and *Gbp^Chr3^* knockout (*Gbp^Chr3^*-KO) BMDMs than in wild-type macrophages has been associated with the ability of GBPs to inhibit the host Arp2/3-dependent actin polymerization machinery and, consequently, *Burkholderia* actin tail formation (33). A similar mechanism has been reported in human cell lines infected with Shigella flexneri, in which the hierarchical targeting of GBPs on the bacteria, reliant on GBP1, impairs *Shigella* actin-based motility, delaying its spread (16, 39, 41). Parting ways with the literature, we did not observe any impairment in the polymerization of the *Burkholderia* actin tails upon GBP targeting in human cells, confirming that the main function of GBPs in response to B. thailandensis infection is to serve as a signaling platform for caspase-4 recruitment and activation. Furthermore, *GBP1*-deficient cells show a level of MNGC formation similar to those of *CASP4*- and *GSDMD*-deficient cells, arguing that in human cells, GBPs restrict replication via pyroptosis induction and not by additional inflammasome-independent mechanisms. It is possible that this discrepancy results from species-dependent differences since additional IFN-induced factors that are not expressed in human epithelial cells might account for the restriction of *Burkholderia* actin dynamics.

In summary, our study is the first to report that interferon restricts the multinucleation events induced by B. thailandensis through the pyroptosis of infected cells. It is important to keep in mind, though, that B. thailandensis is less pathogenic than other species of the B. pseudomallei complex that cause severe disease in humans (1–4). Therefore, further studies are needed to understand whether interferon improves the clearance of *Burkholderia* species that are most adapted to infect humans or whether more-pathogenic species have found ways of escaping GBP/inflammasome-mediated immune surveillance.

## MATERIALS AND METHODS

### Bacterial and mammalian cell culture.

All bacteria were grown at 37°C in an orbital shaker. B. thailandensis strain E264 and its isogenic strain expressing mCherry2 were kindly provided by Marek Basler (Biozentrum, Basel, Switzerland) and were grown in lysogeny broth (LB) medium supplemented with 5 g/liter NaCl. Shigella flexneri M90T expressing the adhesin AfaI was provided by Jost Enninga (Institut Pasteur, Paris, France) and was grown in tryptic soy broth (TSB) supplemented with ampicillin (50 μg/ml). Wild-type HeLa (ATCC CCL-2) and CRISPR-Cas9 knockout HeLa cell lines, generated as previously described (17), were cultured in Dulbecco’s modified Eagle’s medium (DMEM; Gibco) supplemented with 10% fetal calf serum (FCS; BioConcept). HaCaT cells, obtained from CLS (Cell Lines Service) GmbH, were grown in RPMI 1640 (Gibco) supplemented with 10% FCS. HBEC3-KT cells (ATCC) were grown in bronchial/tracheal epithelial cell growth medium (Cell Applications, Inc.). Human primary monocyte-derived macrophages (hMDMs) were purified from buffy coats obtained from the Swiss Red Cross and cultured as described previously (45). Cells were grown at 37°C with 5% CO_2_.

### Infection assays.

When indicated, cells were primed for 16 h with human IFN-γ (Peprotech) at a concentration of 10 ng/ml for HeLa cells and hMDMs or 2.5 ng/ml for HBEC3-KT and HaCaT cells. B. thailandensis cultures grown overnight were adjusted to an optical density at 600 nm (OD_600_) of 1, subcultured 1:20, and grown until mid-exponential phase (OD_600_ = 0.4 to 0.6). S. flexneri cultures grown overnight were subcultured 1/100 and grown until mid-exponential phase (OD_600_ = 0.4 to 0.6). Before infection, bacteria were collected by centrifugation, washed, and resuspended in Opti-MEM (Gibco). Bacteria were added to confluent cells in 96-well plates (HeLa, 5 × 10^4^ cells/well; HBEC3-KT, 2.5 × 10^4^ cells/well; HaCaT, 1 × 10^5^ cells/well; hMDMs, 8 × 10^4^ cells/well) at different multiplicities of infection (MOIs), as described in the figure legends. For B. thailandensis infections, plates were then centrifuged at 300 × *g* for 5 min at 37°C and incubated for 1 h at 37°C. For S. flexneri infections, plates were just incubated at 37°C for 30 min. Noninternalized bacteria were then removed by washing cells three times with prewarmed medium, and cells were incubated with Opti-MEM containing 250 μg/ml kanamycin, in the case of B. thailandensis infections, or 100 μg/ml gentamicin, in the case of S. flexneri infections, in order to kill extracellular bacteria. At the desired time points postinfection (p.i.), cells were either processed for CFU analysis (CFU), multinucleated giant cell (MNGC) quantification, propidium iodide (PI) uptake, or Western blot analysis or fixed for immunofluorescence assays. To determine CFU, infected cells were gently washed with phosphate-buffered saline (PBS) and lysed with water containing 0.2% Triton X-100 at the indicated time points. Bacteria were then serially diluted and plated onto LB agar.

### MNGC quantification assay.

Starting at 20 h p.i., HeLa, HBEC3-KT, and HaCaT cells were stained with Hoechst stain (1:1,000) and examined by fluorescence microscopy. The extent of multinucleation was measured by counting nuclei in 6 fields of view under each experimental condition using Fiji software.

### Plasmids.

Plasmids expressing N-terminally fluorescently tagged GBPs were generated by inserting the GBP coding sequences at the XhoI/HindIII sites of pEGFP-C1 (Clontech) (17). Doxycycline-inducible eGFP and doxycycline-inducible mCherry plasmids were generated by amplifying eGFP and mCherry generated as described above by PCR and inserting the coding sequences at the BamHI site of the pLVX-Puro vector (Clontech). Plasmids expressing LifeAct-eGFP were generated by amplifying eGFP from pLJM1-eGFP (Addgene) and inserting the sequence into the NheI and BstBI cloning sites of a LifeAct-iRFP670 (Addgene) plasmid. All cloning was performed using In-Fusion cloning technology (Clontech), and plasmids were verified by sequencing. When required, HeLa cells were transfected with expression plasmids as previously described (17).

### Lentiviral particle production and HeLa cell transduction.

Lentiviral particles were produced by transfecting HEK293T cells. Cells seeded into a 6-well plate at a density of 1 × 10^6^ cells/well 24 h prior to transfection were transfected with expression plasmids (pLVX-GFP and pLVX-mCherry), packaging plasmid psPax2 (1.9 mg), and envelope plasmid pVSV-G (0.2 mg) using jetPRIME (Polyplus), according to the manufacturer’s instructions. After a 24-h incubation, HEK293T medium containing lentiviral particles was transferred to HeLa cells seeded at a density of 0.8 × 10^6^ cells/well in a 6-well plate. HeLa cells were centrifuged at 2,900 rpm for 90 min and incubated for 48 h (medium was changed after incubation overnight). Puromycin (5.0 μg/ml; InvivoGen) was added to the medium for 6 to 8 days in order to positively select transduced cells.

### Microscopy, time-lapse imaging, and image analysis.

Fluorescence and phase-contrast images of nonfixed samples were obtained using a Leica DFC3000G instrument (40× objective) for MNGC quantification. For fluorescence microscopy of fixed samples, infected HeLa cells and hMDMs were washed twice with PBS and fixed for 20 to 30 min with 4% paraformaldehyde (Electron Microscopy Sciences). Cells were washed four times with PBS and incubated with Hoechst stain (1:1,000) and, when indicated, with CellMask green actin tracking stain (catalog number A57243; Thermo Fisher Scientific) to label F-actin. For ASC speck formation assays, hMDMs were permeabilized with 0.05% saponin and blocked with 1% bovine serum albumin (BSA). Coverslips were then incubated with anti-ASC antibody (catalog number sc-22514-R; Santa Cruz Biotechnology) (1:1,000), washed four times with PBS, and incubated with Hoechst stain (1:1,000). Samples were then analyzed by confocal microscopy by imaging with a Zeiss LSM800 confocal laser scanning microscope using a 63×/1.4-numerical-aperture (NA) oil objective by acquiring Z-stacks with a 300-nm step size. For live imaging, HeLa cells plated onto 8-well μ-slides (Ibidi) at a density of 1 × 10^5^ cells/well were infected as described above. Extracellular B. thailandensis bacteria were removed by washing with warm Opti-MEM, and time-lapse microscopy of living cells was performed in Opti-MEM supplemented with kanamycin (250 μg/ml) at 37°C using a motorized *xyz* stage with autofocus. Samples were imaged with a Zeiss LSM800 confocal laser scanning microscope using a 63×/1.4-NA oil objective by acquiring Z-stacks with a 600-nm step size. Data were further analyzed and processed using Fiji software, and all fluorescence-derived images shown correspond to maximum three-dimensional (3D) projections.

### siRNA-mediated knockdown.

HaCaT (5 × 10^4^ cells/well) and HBEC3-KT (2.5 × 10^4^ cells/well) cells were seeded into a 96-well plate and transfected with 25 nM or 30 nM Stealth RNAi siRNAs (Thermo Fisher Scientific) using Lipofectamine RNAiMax (Thermo Fisher Scientific). By 7 to 8 h posttransfection, cells were primed with human IFN-γ for 16 h and then infected as described above. Phase-contrast images of siRNA knockdown cells were acquired at 20 to 24 h p.i. to assess MNGC formation. The siRNA-mediated knockdown effectiveness was tested by Western blot analysis. The siRNAs used are as follows: the siRNA negative control, indicated as nontargeting (NT) siRNA (catalog number 12935300; Thermo Fisher Scientific); siRNA targeting CASP4 (siCASP4) (catalog number HSS141457; Thermo Fisher Scientific); siGSDMD (catalog number HSS149278; Thermo Fisher Scientific); and siGBP1 (catalog number HSS104021; Thermo Fisher Scientific).

### PI uptake and Western blot analysis.

Cell permeabilization was quantified by measuring PI uptake. PI (Thermo Fisher Scientific) was added to the medium at 12.5 μg/ml, and fluorescence was measured over time using a Cytation5 plate reader (BioTek). To account for spontaneous cell permeabilization, PI uptake was normalized to 100% lysis and the uninfected control. Western blot analysis was performed by lysing cells in 1× sample buffer (Thermo Fisher Scientific) with the addition of 66 mM Tris-Cl (pH 7.4), 2% SDS, and 10 mM dithiothreitol (DTT). In assays where we checked caspase-4 processing by Western blotting, cell lysates were combined with precipitated supernatants. Samples were boiled for 5 min at 95°C, and proteins were separated using 12% SDS-PAGE. Proteins were then transferred onto 0.2-μm polyvinylidene difluoride (PVDF) membranes using the Trans-Blot Turbo system (Bio-Rad). Membranes were blocked in a solution of Tris-buffered saline–Tween (TBS-T) with 5% milk and incubated with primary antibody, followed by incubation with horseradish peroxidase (HRP)-coupled secondary antibodies. Western blot membranes were analyzed by Fusion Solo S (Vilber) using the Pierce ECL Western blotting substrate (Thermo Fisher Scientific) or the Pierce ECL Plus Western blotting substrate (Thermo Fisher Scientific). The antibodies employed are as follows: mouse anti-caspase-4 clone 4B9 (catalog number ADI-AAM-114-E; Enzo Life Science) (1:750), rabbit anti-GSDMD (catalog number ab210070; Abcam) (1:1,000), rabbit anti-GSDMD (catalog number CSB-PA009956GA01HU; Cusabio) (1:1,000), rabbit anti-GBP1 (catalog number ab131255; Abcam) (1:1,000), mouse anti-alpha-tubulin-HRP conjugate (catalog number ab40742; Abcam) (1:1,000), goat anti-rabbit IgG-HRP (catalog number 4030-05; Southern Biotech) (1:5,000), and goat anti-mouse IgG-HRP (catalog number 1034-05; Southern Biotech) (1:5,000).

### Data analysis.

Data analysis was performed using Gen5, GraphPad Prism v9, Microsoft Excel, and Fiji software. Statistical significance is indicated as *, **, or *** for a *P* value of <0.05, <0.01, or <0.001, respectively.
