# Erratum for Dilucca et al., “Guanylate-Binding Protein-Dependent Noncanonical Inflammasome Activation Prevents Burkholderia thailandensis-Induced Multinucleated Giant Cell Formation”

**DOI:** 10.1128/mbio.00694-23

**Published:** 2023-04-11

**Authors:** Marisa Dilucca, Saray Ramos, Kateryna Shkarina, José Carlos Santos, Petr Broz

## ERRATUM

Volume 12, no. 4, e02043-21, 2021, https://doi.org/10.1128/mBio.02054-21. Supplemental material, Movies S1 to S7: The movie files are out of order. Specifically, the file called Movie S1 is Movie S2, the file called Movie S2 is Movie S3, the file called Movie S3 is Movie S4, the file called Movie S4 is Movie S5, the file called Movie S5 is Movie S6, the file called Movie S6 is Movie S7, and the file called Movie S7 is Movie S1. The movie legends are correct.

