# Supplementary figures and images for: Guanylate-Binding Protein-Dependent Noncanonical Inflammasome Activation Prevents Burkholderia thailandensis-Induced Multinucleated Giant Cell Formation
Source: mBio. 2021 Aug 17;12(4):e02054-21. doi: 10.1128/mBio.02054-21 (PMC8406320; doi:10.1128/mBio.02054-21)

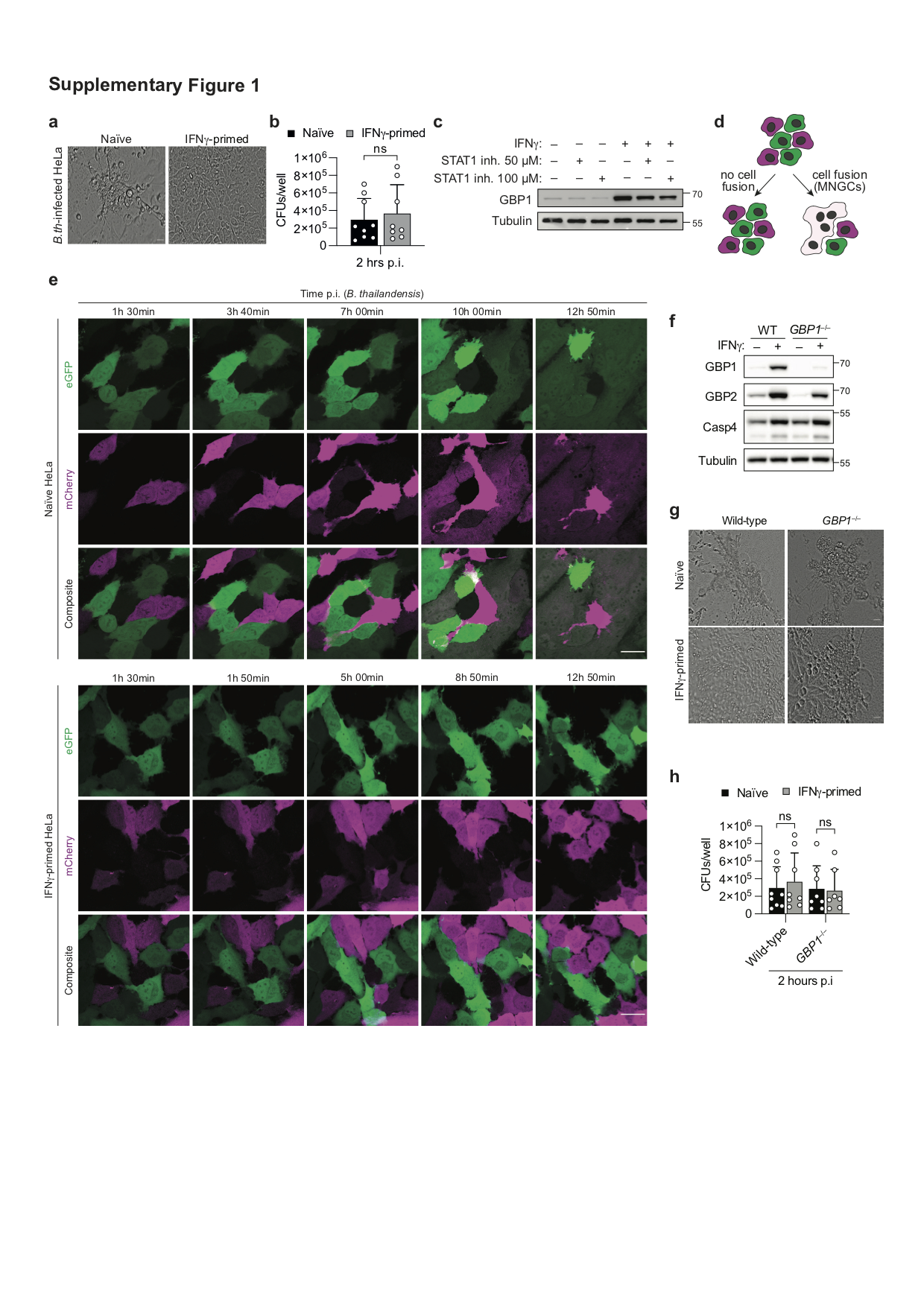

Supplement: FIG S1 [file mbio.02054-21-sf001.tif]

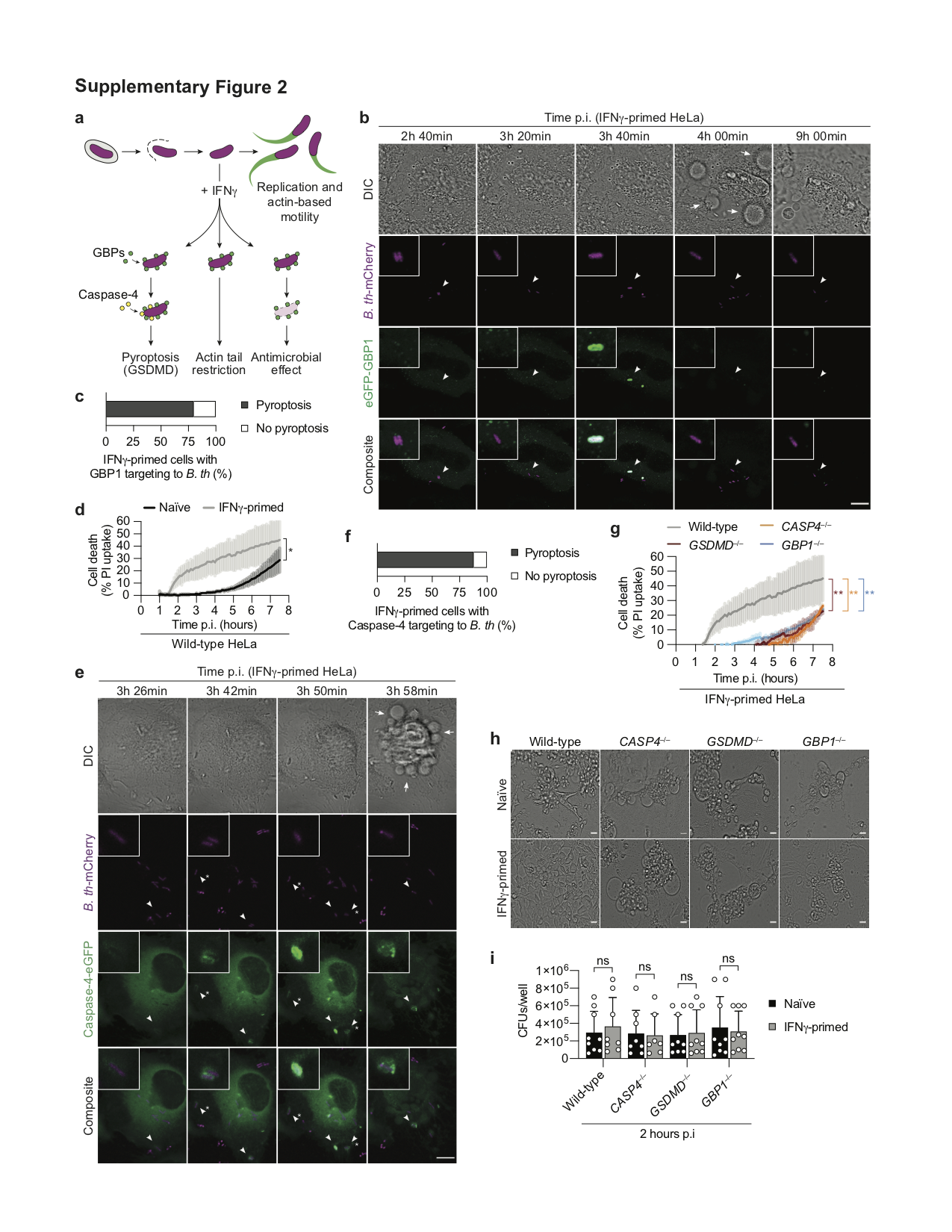

Supplement: FIG S2 [file mbio.02054-21-sf002.tif]

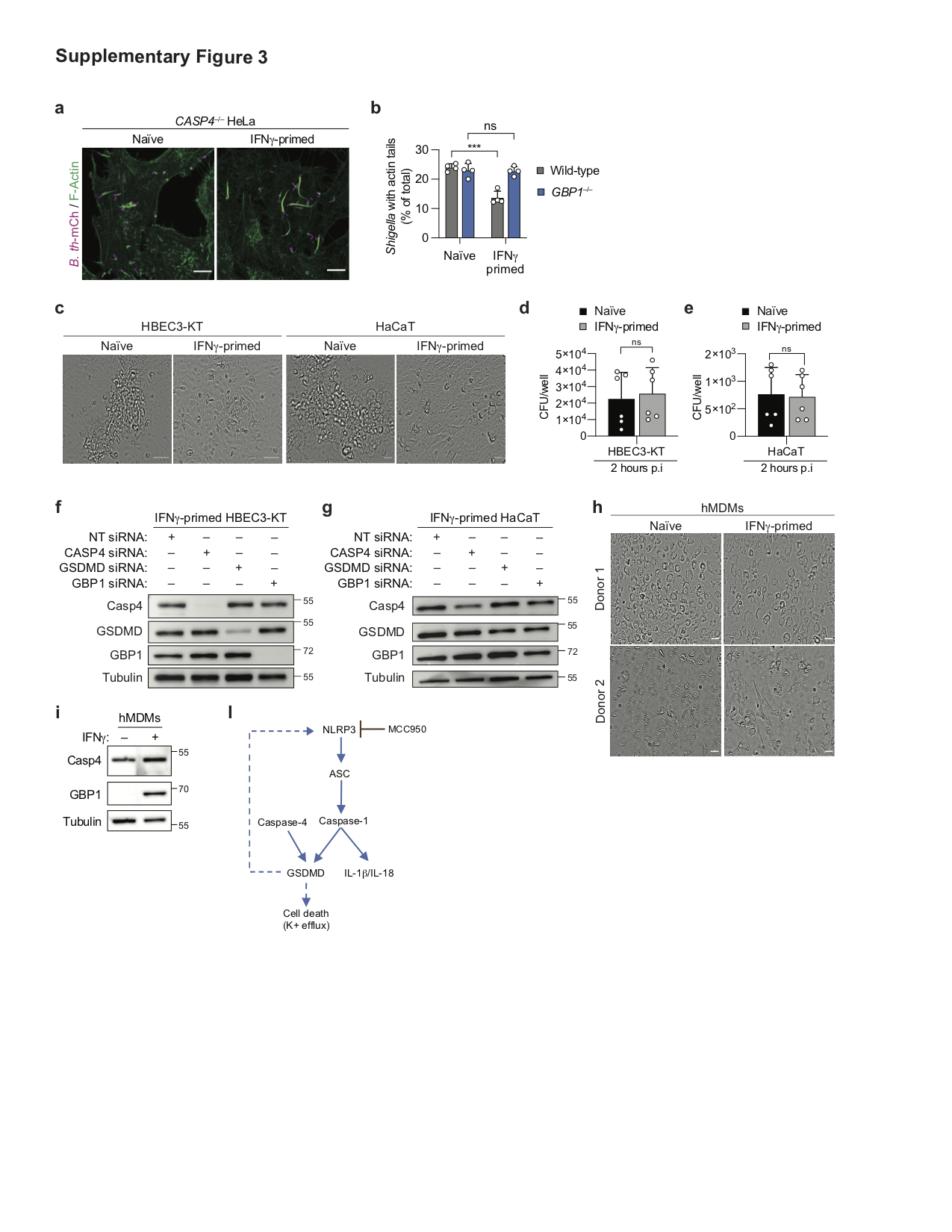

Supplement: FIG S3 [file mbio.02054-21-sf003.tif]
